# Bridging the Gap between the Lab and the Clinic: Psychopathology's Grand Challenge

**DOI:** 10.3389/fpsyg.2016.01752

**Published:** 2016-11-08

**Authors:** Xavier Noël, Antoine Bechara

**Affiliations:** ^1^Laboratoire de Psychologie Médicale et d'Addictologie, Université Libre de BruxellesBrussels, Belgium; ^2^Department of Psychology and Brain and Creativity Institute, University of Southern CaliforniaLos Angeles, CA, USA

**Keywords:** psychopathology, aetiology, development, brain-culture, taxonomy

Psychopathology is the scientific exploration of clinically significant disturbances in an individual's cognition, emotion regulation, or behavior, which reflect a dysfunction in the psychological, biological, or developmental processes underlying mental functioning [American Psychiatric Association (APA), 2013]. The past two decades witnessed an accumulation of evidence for “psychopathological states as brain disorders,” which ignited new hopes that neuroscience will contribute to the advancement of clinical practices in psychopathology (Ekhtiari and Paulus, 2016). However, these hopes were met with a few hurdles that must be overcome because translating basic knowledge about the neural mechanisms behind several psychiatric conditions to a clinical application is not very simple. Some of the reasons for these encountered difficulties to “*bridge the gap between lab and clinic”* are numerous and can be divided into four categories: (1) the lack of direct connections between basic research and clinical conditions; (2) a tunnel vision that is too focused on the brain itself, which often ignores the contextual and historical (developmental) influences of the clinical condition under study; (3) the lack of consideration for individual differences in a given clinical population; and (4) the questionable validity of using a categorical mental disorder diagnosis. Therefore the “*here and now”* approach in psychopathology is not sufficient, and it should complement an approach that considers the disease process over time (Heckers, 2014).

## About the resistance to translational research in behavioral analysis

The first challenge is improving the collaboration between basic and pre-clinical scientists on one hand, and the practicing clinicians on the other. Indeed, several decades ago, when clinical psychologists and psychiatrists were asked to rank the usefulness of research articles and scientific books to their clinical practice, the ranking fell near the bottom of the scale (Weisz et al., 1995). Unfortunately, this situation has not improved very much since that time. Therefore increasing some of the lab-clinic interactions are needed for psychopathology research. For instance, laboratory procedures (e.g., attentional training in the context of anxiety disorders) cannot be brought to clinical practice without significant adjustments to fit the clinical environment. Similarly, clinicians communicating with lab researchers may inform scientists on clinical outcomes, and the clinical phenomena encountered in their therapy. Unfortunately these interactions are often lacking because specialists, such as clinical psychologists and psychiatrists, tend to adhere to the protocols of their own area, and they could be less open minded about the practicess of another area. The same can be said about lab researchers who are often less interested in translational research and clinical applications. This adherance to one's specialty tends to compromise the necessary lab/clinic interactions for making adjustemnts and for generating new ideas. Indeed, while clinical outcome studies can provide information about the overall efficacy of a treatment, they gather too limited information about the mechanisms of therapeutic change. Translational research can help identify not only what works in interventions aimed at faciliting emotional, cognitive and behavioral changes, but also how it produces positive results. Some of these interactions are beginning to take place, thus resulting in relevant theoretical contributions (e.g., on how an expansion of the alexithymia construct stimulates new forms of mechanisms-based clinical interventions (Lane et al., 2015).

Indeed, the field of psychopathology has gained considerably from encouraging interdisciplinary and translational research. However, one of the reasons for the hurdles encountered in bridging the gap between basic science and the clinic is the much too narrow brain-oriented research in psychopathology, which tends to focus on underlying brain mechanisms without much consideration for the environmental influences of this condition. A better approach would consider the brain and environment as two dynamic forces with constant mutual influences.

## A tunnel vision that is too focused on the brain itself

Another reason for the challenge of “bridging the gap” is the lack of consideration for individual variability, and the notion that not every individual with some abnormal behavior is necessarily “diseased.” Indeed, one intriguing issue to consider is how social representations of a mental disease could shape the brain in different ways. Far from being a universal constant, brain processes underlying psychological states are shaped by culture [e.g., see the emerging field on cultural neuroscience (Chiao and Immordino-Yang, 2013)]. More specifically, there is a risk in “over-pathologizing” everyday life behaviors, and in focusing on the “pathological” aspects of the behavior without considering other “healthy” processes that help correct the abnormal behavior. For example, consider the view of alcoholism according to the “disease” model. This view considers addiction as an irreversible deterioration of neural processes serving affective and cognitive functioning. Although, it has been massively documented that addiction changes the brain just like diabetes changes the way the pancreas works, thus dramatically disturbing the normal hierarchy of needs and desires, it is noteworthy seriously consider evidence from studies on self-change, which is also referred to as natural recovery (Slutske, 2006). Proponents of the natural recovery model argue that addicted individuals would be less prone to engage themselves into a self-change strategy if the societal response to addiction problems and views are based exclusively on a “disease model.” A self-change-friendly society would focus also on the protective factors that promote the resilience of the individual against alcoholism, instead of focusing only on deficits. This is also relevant to the brain approach to psychopathological disorders because it indicates that self-regulatory brain mechanisms may be impacted heavily by addiction policies and images of afflicted individuals in the population. Another issue is the assumption, which is mostly based on the *pure* neurological syndrome, that when the brain psychopathology is so severe, then a brain deficit is implicated, and looking at environmental changes as a form of treatment might be less effective. Perhaps the future of psychopathology research should seek better understanding of the relationship between brain, context, and history, which should lead to more comprehensive and satisfactory explanations necessary for innovative treatments.

## Focus on individual differences in a given clinical population

A third reason for the challenge of “bridging the gap” is the questionable validity of using a categorical mental disorder diagnosis. A good deal of knowledge in the past several years came from the accumulating evidence that psychopathological conditions are associated with multiple causes (i.e., the concept of equifinality). Indeed, a given state can be reached by many potential means and psychopathological states exemplify this principle. For this reason, an important clinical approach that became popular is the “*What works for whom*?” approach. The clinical course of the mental problem to be treated, the developmental pathways, the genetic susceptibilities and environmental protective and risk factors have all urged clinical scientists to customize their clinical interventions. Tailoring interventions to individuals may result in improving the efficiency of treatment delivery and maximizing its impact. Indeed, although efficient, therapeutic interventions in humans are at best relatively weak (see for instance Cohen's criterion). Failure to consider inter-individual variation in developmental pathways and past or present living context means that, for some individuals, intervention efficacy could be much higher, and much smaller for others.

## On the questionable validity of using a categorical mental disorder diagnosis

Finally, in an effort to develop alternative classification methods to the symptom-based diagnostic systems such as the *Diagnostic and Statistical Manual of Mental Disorders (DSM)* and the *International Classification of Diseases (ICD)*, the NIMH encouraged researchers to take a dimensional approach to the study of the genetic, neural, and behavioral features of mental disorders (Morris and Cuthbert, 2012). The justification for this effort comes from the evidence that the issues of comorbidity (meeting one criteria for one disorder augments the probability to be diagnosed with another), the heterogeneity of symptoms (two persons with the same diagnosis look very different), and the biological specificity are unacceptably low when using the older approach of drawing categorical lines between disorders. The development of the Research Domain Criteria consists of a matrix where the rows represent specified functioning constructs (e.g., genes, molecules, circuits) grouped into higher-level domains of functioning (emotion, cognition, motivation and social behavior). The primary domains under studies include valence systems (positive, negative), cognitive systems (attention, memory, cognitive control, etc.), systems for social processing (affiliation and attachment, social communication, perception of self, etc.), arousal/regulatory systems (circadian rhythms, sleep). This paradigm shift looks very promising as it offers new perspectives regarding the relationships between brain and behavior in mental illnesses, including psychopathology.

As an initial sign of addressing these outlined issues, many scientists are now considering the use of a variety of “big data” methods. One example is the IMAGEN multi-centre project (Schumann et al., 2010), which collects diagnostic, cognitive, genetic, contextual, and neural data of thousands of 14-year-old adolescents over time with the aim of identifying predictors of mental health and risk taking behavior. Another example is INTERNET testing(Gillan and Daw, 2016) that allows collecting large samples of data, which in turn could provide a tremendous potential for psychiatric research.

In sum, *bridging the gap* in psychopathology necessitates overcoming a number of hurdles, which we outlined. Addressing these four outlined difficulties should stimulate future research in several directions. This is what *Frontiers in Psychopathology* hopes to achieve. Exploring the translational pathway between research in neuroscience and conceptually novel forms of therapeutic interventions is of great value. Research on how abnormalities across brain-body-environment systems contribute to the vulnerabilities in psychopathology is seen as critical. Indeed, complementing to the so-called “*encephalocentrism,”* the exploration of how organism's sensory-motor experience in relation to its environment has the potential to advance our comprehensive models of psychopathological states (Fuchs and Schlimme, 2009; Harshaw, 2015). The recent recognition that the insula, responsible for conscious accessible feelings from “*raw”* somatic signals (Craig, 2009) is among the regions most frequently associated with psychopathological conditions (e.g., craving determinants of smoking addiction; Naqvi et al., 2007) should encourage the research on embodiment in psychopathology. *Frontiers in Psychopathology* also welcomes studies that investigate inter-individual differences in a given clinical population and inter-individual responses to clinical trials, multi-dimensional longitudinal approaches affording a more valid identification of causes and effects related to mental illnesses, and importantly, the influence of cultural, and society on the expression of mental illnesses.

## Author contributions

XN and AB contributed equally to this work.

## Funding

XN is supported by the Fonds de la Recherche Scientifique (F.R.S./FNRS).

### Conflict of interest statement

The authors declare that the research was conducted in the absence of any commercial or financial relationships that could be construed as a potential conflict of interest. The handling Editor declared a shared affiliation, though no other collaboration, with one of the authors XN and states that the process nevertheless met the standards of a fair and objective review.
